# Clinical implications of seropositive and seronegative autoantibody status in rheumatoid arthritis patients: A comparative multicentre observational study

**DOI:** 10.1515/rir-2024-0007

**Published:** 2024-03-31

**Authors:** Nevin Hammam, Passant N. El-Husseiny, Suzan S. Al-Adle, Nermeen Samy, Nora Y. Elsaid, Dina F. El-Essawi, Eman F. Mohamed, Samar M. Fawzy, Samah A. El Bakry, Maha Nassr, Samah I. Nasef, Hanan M. El-Saadany, Shereen Elwan, Nada M. Gamal, Abdelhfeez Moshrif, Osman Hammam, Rawhya R. El Shereef, Faten Ismail, Samar Tharwat, Doaa Mosad Mosa, Mervat I Abd Elazeem, Enas A. Abdelaleem, Tamer A. Gheita, on behalf of The Egyptian College of Rheumatology (ECR) Rheumatoid Arthritis (ECR-RA) Study Group

**Affiliations:** Rheumatology Department, Faculty of Medicine, Assiut University, Assiut, Egypt; Rheumatology Department, Faculty of Medicine, Cairo University, Cairo, Egypt; Internal Medicine Department, Rheumatology Unit, Faculty of Medicine, Ain-Shams University, Cairo, Egypt; Health Radiation Research Department, Internal Medicine Unit, Rheumatology Clinic, (NCRRT), Egyptian Atomic Energy Authority (EAEA), Cairo, Egypt; Internal Medicine Department, Rheumatology Unit, Faculty of Medicine (Girls), Al-Azhar University, Cairo, Egypt; Rheumatology Department, Faculty of Medicine, Fayoum University, Fayoum, Egypt; Rheumatology Department, Faculty of Medicine, Suez-Canal University, Ismailia, Egypt; Rheumatology Department, Faculty of Medicine, Tanta University, Gharbia, Egypt; Rheumatology Department, Faculty of Medicine, Al-Azhar University, Assiut, Egypt; Department of Rheumatology and Rehabilitation, Faculty of Medicine, New Valley University, New Valley, Egypt; Rheumatology Department, Faculty of Medicine, Minia University, Minia, Egypt; Internal Medicine Department, Rheumatology Unit, Faculty of Medicine, Mansoura University, Dakahlia, Egypt; Department of Rheumatology and Rehabilitation, Mansoura University Hospitals, Mansoura University Faculty of Medicine, Mansoura, Egypt; Rheumatology Department, Faculty of Medicine, Beni-Suef University, Beni-Suef, Egypt

**Keywords:** anti-cyclic citrullinated peptide, egyptian college of rheumatology, rheumatoid arthritis, rheumatoid factor

## Abstract

**Background and Objectives:**

Rheumatoid factor (RF) and anti-cyclic citrullinated protein (anti-CCP) have been used to improve the diagnosis and prognosis of rheumatoid arthritis (RA). However, their association with RA disease phenotypes, individually and in combination, is not well studied. The aim of the study was to compare patients’ and disease characteristics, activity and severity in double seronegative (DNRA), single seropositive RF, single seropositive anti-CCP and double seropositive (DPRA) patients.

**Methods:**

Adults subjects with RA from Egyptian College of Rheumatology (ECR) database who had RF and anti-CCP results available were included. Demographic, clinical features, disease activity score 28 (DAS28), Health Assessment Questionnaire (HAQ) and laboratory data were collected and compared among different RA groups.

**Results:**

5268 RA patients with mean age of 44.9±11.6 years, and 4477 (85%) were females. 2900 (55%) had DPRA, 892 (16.9%) had single positive RF, 597 (11.3%) had single positive anti-CCP while 879 (16.7%) had DNRA. Patients with DPRA had significantly high percentage of metabolic syndrome (19.3%, *P* < 0.001), and functional impairment using HAQ (*P* = 0.01). Older age (RRR [relative risk ratio]: 1.03, 95%CI: 1.0, 1.0, *P* = 0.029), greater DAS28 (RRR: 1.51, 95%CI: 1.2, 1.9, *P* < 0.001), higher steroid use (RRR: 2.4, 95%CI: 1.36, 4.25, *P* = 0.002) were at higher risk of DPRA while longer disease duration (RRR: 1.08, 95%CI: 1.01, 1.16, *P* = 0.017) and fibromyalgia syndrome (RRR: 2.54, 95%CI: 1.10, 5.88, *P* = 0.028) were associated with higher odds of single positive RF status.

**Conclusion:**

Dual antibody-positive status has higher disease activity and severity, and higher chance of development of metabolic syndrome; highlighting the implicated role of inflammation, atherogenesis and cardiovascular disease risk in RA.

## Introduction

Rheumatoid arthritis (RA) is a chronic inflammatory autoimmune disease characterized by arthritis and subsequent destruction.^[1]^ The diverse spectrum of RA is associated with female predominance.^[2]^ RA is characterized by the production of autoantibodies, rheumatoid factor (RF) and anti-cyclic citrullinated protein (anti-CCP), that provide clues to the underlying disease pathophysiology. RF, an autoantibody directed against the Fc part of IgG, is an important serological marker for the diagnosis of RA in everyday clinical practice. During the last decade, multiple antibodies have been identified in RA against the citrullinated peptides including IgG, IgA, IgM.^[3]^ Anti-CCP has been emerging as a highly specific marker in RA diagnosis and prognosis.^[4]^ Both tests are sensitive; however, anti-CCP is more specific for RA diagnosis.^[5]^ The presence of RF positivity or anti-CCP is incorporated and weighed in the 2010 American College of Rheumatology/European League Against Rheumatism (ACR/EULAR) classification criteria for RA.^[6]^

Researchers have confirmed the significance of RF and anti-CCP for the early diagnosis of RA^[7]^ and disease activity evaluation.^[8]^ Despite this rising importance of autoantibodies in RA, there has been substantial discrepancy whether seropositive RA has more severe disease spectrum compared to seronegative RA. Higher disease activity and impaired physical function in double seropositive RA (RA and anti-CCP) at baseline, in addition to more radiographic progression has been described.^[9]^ In contrast, others reported severer activity^[10]^ and radiographic progression in seronegative RA.^[11]^ Bizzaro et al.^[12]^ found that anti-CCP levels were highly related to early RA but not to age and gender. Egyptian RA patients with high titers of RF and anti-CCP were associated with impairment in the physical function and higher steroid doses compared to those with low titers.^[13]^

Both of these antibodies, singly or in combination, may be representatives of different RA specific diseases features and drug responsiveness. Thus, anti-CCP and RF are providing potential different RA disease phenotypes. The aim of the current work was to assess the frequency of positive RF and anti-CCP singly or in combination, and to explore the clinical profile (demographic factors, disease characteristics, activity and severity, and laboratory features) associated with each antibody status RA groups: single-positive RF, single-positive anti-CCP, double-positive RF+/anti-CCP+ (DPRA) and double negative RA (DNRA). Factors associated with seropositive status were also well thought out.

## Patients and Methods

This study is a multicentre observational study of the Egyptian College of Rheumatology-RA study group (ECR-RA) study, which has been described in detail elsewhere.^[2]^ Adult patients with RA were recruited between September 2018 till December 2021. Data were extracted from the ECR-RA database for patients with diagnosis of RA (*N* = 10, 364) confirmed using the 2010 ACR/EULAR classification criteria.^[6]^ Patients with missing RF or anti-CCP data were excluded (*N* = 5096). Patients provided informed consents to participate, and the ECR-RA study was approved by the Scientific Research and Ethical Committee and in accordance to the 1964 Helsinki declaration.

All patients were subjected to full history taking and clinical examination including body mass index (BMI). Patients’ demographic data including age, sex and disease characteristics as well as systems involved were collected. The major clinical features of RA including arthritis, mucous ulcer, ocular features (keratoconjunctivitis sicca, episcleritis, scleritis), neurologic manifestations (depression, peripheral neuropathy), vasculitis (cutaneous, system vasculitis), gastrointestinal manifestations (abdominal pain, hepatomegaly, elevated transaminase, autoimmune hepatitis), cardiovascular manifestations (pericarditis/pericardial effusion, myocarditis, endocarditis, valvular insufficiency), chest manifestations (pleurisy/pleural effusion, pulmonary infiltrates), and renal manifestations (proteinuria, hematuria, elevated serum creatinine).

The disease activity score-28 (DAS-28)^[14]^ and health assessment questionnaire (HAQ)^[15]^ were assessed. Comorbidities, such as diabetes mellitus, hypertension, metabolic syndrome (MetS), hepatitis C virus (HCV), thyroid dysfunction and bronchial asthma were recorded. Current medications use including synthetic disease modifying anti-rheumatic drugs (DMARDs), in addition to steroids were recorded. The laboratory investigations including complete blood count (CBC), liver and kidney function tests, erythrocyte sedimentation rate (ESR), C-reactive protein (CRP), and lipid profile panel were recorded. RF and anti-CCP presence were determined.

Patients were grouped according to the autoantibodies (RF and anti-CCP) status; DNRA was defined as absence of both RF and anti-CCP; DPRA was defined as presence of both RF and anti-CCP, single-positive RF and single-positive anti-CCP.

### Statistical analysis

It was performed using Stata statistical software version 15 (Stata-Corp). Descriptive analyses were performed. Continuous variables were expressed as means (standard deviations), or medians (interquartile range). Categorical variables were expressed as absolute numbers and percentages. Patients in different groups were compared using One-way analysis of variance (ANOVA) or chi-square as appropriate, followed with pairwise comparisons of means to compute all differences of means of variables between individual groups. Multinomial logistic regression analysis was performed to determine independent variables that would predict single and double positivity autoantibody status. Results are summarized as relative risk ratios (RRR) and confidence intervals (CI). Two-sided p-values were considered significant at < 0.05.

## Results

The characteristics and comparisons between each RA subgroups according to RF and anti-CCP status are presented in tables 1, 2, 3, and 4. A total of 5268 patients from the ECR-RA database were included in the study, mean age was 44.9±11.6 years, and 4477 were females (85%) and 791 were males (15%). The mean DAS28 and HAQ scores were 4.4±1.4 and 0.9±0.6 respectively. 2900 (55%) had DPRA, 892 (16.9%) had single positive RF, and 597 (11.3%) single positive anti-CCP. Both antibodies were negative in 879 (16.7%) of the patients.

Patients with single positive anti-CCP were younger (42.5 years) compared to other groups (*P* < 0.001). Female: male ratio was varied between groups; however, the frequency of males was significantly higher in DPRA (*P* = 0.001). Smoking was significantly more frequent in single positive RF group (*P* = 0.006). Further demographic characteristic information for each RA group is provided in Table 1.

**Table 1:** Characteristics of the rheumatoid arthritis patients and comparison of different groups regarding demographic, and comorbidities according to autoantibodies status.

Parameter Mean ±SD or *n* (%)	Rheumatoid arthritis patients					

			Seropositive rheumatoid arthritis (*n* = 4389)			
	
	All (*n* = 5268)	DNRA (*n* = 879)	DPRA (*n* = 2900)	RF+/anti-CCP- (*n* = 892)	anti-CCP+/RF- (*n* = 597)	*P* value
*Demographics*						
Age (years)	44.9 ± 11.6	43.8 ± 12.2	45.6 ± 11.3^a^	45.2 ± 12^a^	42.5 ± 10.9^b,c^	<0.0001
Female	4477 (85)	770 (87.6)	2416 (83.3)^a^	764 (85.7)	527 (88.3)^b^	0.001
Body mass index	28.6±5.3	28.4 ± 5.6	28.8 ± 5.2	28.4 ± 5.3^a^	27.8 ± 5.5^b^	0.02
Smoking	402/3625 (11.1)	73/572 (12.8)	218/2076 (10.5)	84/604 (13.9)	27/369 (7.3)^a,c^	0.006
*Comorbidities*						
Diabetes mellitus	418/5038 (8.3)	76/873 (8.7)	229/2701 (8.5)	66/889 (7.4)	47/575 (8.2)	0.8
Hypertension	592/5038 (11.8)	90/873 (10.3)	317/2701 (11.7)	118/889 (13.2)	67/575 (11.7)	0.3
Metabolic syndrome	297/1824 (16.3)	38/265 (14.3)	207/1074 (19.3)	30/324 (9.3)^b^	22/161 (13.7)	<0.0001
HCV infection	37/5038 (0.7)	2/873 (0.2)	16/2701 (0.6)	17/889 (1.9)^a,b^	2/575 (0.4)^c^	<0.0001
Bronchial asthma	26/5038 (0.5)	5/873 (0.6)	13/2701 (0.5)	6/889 (0.7)	2/575 (0.4)	0.8
Thyroid dysfunction	83/5038 (1.7)	14/873 (1.6)	52/2701 (1.9)	12/889 (1.4)	5/575 (0.9)	0.7

DNRA, double seronegative RA; DPRA, double seropositive RA; RF, rheumatoid factor; Anti-CCP, anti-cyclic citrullinated peptide; HCV, hepatitis C virus. Statistically significant *P* values are in bold. ^a^Significantly different from DNRA, ^b^Significantly different from DPRA, ^c^Significantly different from RF+/anti-CCP-.

Tables 1 and 2 lists the significant differences of the most important clinical and laboratory features between each RA groups. Patients with single positive anti-CCP were younger (42.5 years) and had an earlier age of disease onset (36.5 years) compared to other groups (*P* < 0.001). Patients with DPRA had significantly higher frequency of MetS (19.3%, *P* < 0.001), arthritis (68.7%, *P* < 0.001), anemia (68.9%, *P* < 0.0001), and functional impairment (*P* = 0.01). The percentage of patients with ocular (22.3%), central nervous system (12.6%) and cardiovascular disease (14.5%) were significantly more frequent in DNRA (*P* = 0.03, *P* = 0.0006 and *P* = 0.005 respectively). Among discordant antibody status, with only one antibody positive, patients with either positive anti-CCP or RF presented with higher fibromyalgia syndrome (30% and 26.9% respectively, *P* < 0.0001); however, patients with single positive RF presented with higher gastrointestinal diseases (36.2%, *P* < 0.0001) and HCV (*P* < 0.0001).

**Table 2:** Clinical characteristics of rheumatoid arthritis patients and comparison of different groups regarding the clinical characteristics, disease activity and severity according to autoantibodies status

Parameter Mean ±SD or *n* (%)	Rheumatoid arthritis patients					

			Seropositive rheumatoid arthritis (*n* = 4389)			
	
	All (*n* = 5268)	DNRA (*n* = 879)	DPRA (*n* = 2900)	RF+/anti-CCP- (*n* = 892)	anti-CCP+/RF- (*n* = 597)	*P* value
Disease duration	6.37 ± 5.5	5.8 ± 5.5	6.5 ± 5.4 a	6.8 ± 5.8 a	6.03 ± 5 c	<0.0001
Age at onset	38.5 ± 11.1	38.1 ± 11.5	39.2 ± 10.8	38.3 ± 11.9	36.5 ± 10.3^a,b,c^	<0.0001
Arthritis	2339/2859 (81.8)	351/461 (76.1)	1344/1551 (86.7)^a^	419/553 (75.8)^b^	225/294 (76.5)^b^	<0.0001
RA nodules	287/3008 (9.5)	44/446 (9.9)	161/1771 (9.1)	38/449 (8.5)	44/342 (12.9)	0.2
Mucous ulcers	67/1145 (5.9)	13/143 (9.1)	26/681 (4.1)	8/199 (4)^a^	18/122 (14.8)^b,c^	<0.0001
Ocular	571/3118 (18.3)	113/507 (22.3)	285/1710 (16.7)^a^	106/554 (19.1)	67/347 (19.3)	0.03
Sjogren’s syndrome	492/3187 (15.4)	75/449 (16.7)	272/1899 (14.3)	94/488 (19.3)^b^	51/351 (14.5)^b^	0.05
Neurological manifestations	267/2945 (9.1)	52/414 (12.6)	142/1761 (8.1)^a^	52/467 (11.1)	21/303 (6.9)^a^	0.006
Vasculitis	68/2502 (2.7)	10/341 (2.9)	43/1534 (2.8)	6/369 (1.6)	9/258 (3.5)	0.5
Gastrointestinal manifestations	635/2472 (25.7)	96/401 (23.9)	312/1382 (22.6)	163/450 (36.2)^a,b^	64/239 (26.8)^c^	<0.0001
Cardiovascular manifestations	333/3180 (10.5)	76/525 (14.5)	158/1740 (9.1)^a^	63/562 (11.2)	36/353 (10.2)	0.005
Pulmonary manifestations	453/3154 (14.4)	75/462 (16.2)	239/1840 (12.9)	82/507 (16.2)	57/345 (16.5)	0.08
Osteoporosis	37/5038 (0.7)	2/873 (0.2)	24/2701 (0.9)	8/889 (0.9)	3/575 (0.5)	0.2
Fibromyalgia	446/2025 (22)	51/298 (17.1)	219/1101 (19.9)	101/376 (26.9)^a,b^	75/250 (30)^a,b^	<0.0001
Renal manifestations	150/2899 (5.2)	31/454 (6.8)	79/1622 (4.9)	25/506 (4.9)	15/317 (4.7)	0.4
Anemia	873/1389 (62.9)	88/182 (48.4)	630/914 (68.9)^a^	76/140 (54.3)^b^	79/153 (51.6)^b^	<0.0001
DAS28	4.4 ± 1.4	4.4 ± 1.5	4.4 ± 1.4	4.4 ± 1.4	4.3 ± 1.4	0.2
HAQ	0.9 ± 0.6	0.9 ± 0.7	1.0 ± 0.6	0.9 ± 0.6	0.8 ± 0.5^b^	0.01

DNRA, double seronegative RA; DPRA, double seropositive RA; RF, rheumatoid factor; Anti-CCP, anti-cyclic citrullinated peptide; DAS-28, disease activity score28; HAQ, health assessment questionnaire. statistically significant *P* values are in bold. ^a^Significantly different from DNRA, ^b^Significantly different from DPRA, ^c^Significantly different from RF+/anti-CCP-.

The percentage of patients with steroids use was higher among DPRA patients (72.2%, *P* = 0.01); while those with DNRA were most likely to use sulfasalazine (59.6%, *P* < 0.001). Patients with single positive RF were less likely to use methotrexate (69.2%, *P* = 0.002)(Table 4).

**Table 3:** Laboratory Characteristics of rheumatoid arthritis patients and comparison of different groups according to RF and anti-CCP autoantibody status

Parameter Mean ±SD or *n* (%)	Rheumatoid arthritis patients					

			Seropositive rheumatoid arthritis (n=4389)			
	
	All (*n* = 5268)	DNRA (*n* = 879)	DPRA (*n* = 2900)	RF+/anti-CCP- (*n* = 892)	anti-CCP+/RF- (*n* = 597)	*P* value
Hb (g/dL)	11.5 ± 1.4	11.7 ± 1.4	11.4 ± 1.3^a^	11.6 ± 1.5^b^	11.7 ± 1.4^b^	<0.0001
TLC×10^3^ (cell/mm^3^)	6.9 ± 2.3	6.9 ± 2.6	6.9 ± 2.3	6.9 ± 2.3	7.1 ± 2.3	0.5
Platelets×10^3^ (cell/mm^3^)	294.2 ± 92.4	286 ± 83.1	298.2 ± 95.8^a^	288.1 ± 91.3^b^	297.3 ± 90.1	0.004
ESR (mm/1^st^ hour)	44.1 ± 27.9	41.2 ± 25.9	45.8 ± 29.4^a^	45.7 ± 26.4^a^	38.6 ± 24.9^b,c^	<0.0001
Positive CRP	3139/4220 (74.4)	427/597 (71.5)	1896/2490 (76.14)	494/649 (76.12)	322/484 (66.5)^b, c^	<0.0001
ALT (U/L)	24.4 ± 13.9	24 ± 20.9	24.8 ± 12.9	24.3 ± 11.4	23.0 ± 10.8	0.2
AST (U/L)	25.8 ± 13.1	25.3 ± 17.8	26.6 ± 12.6	24.6 ± 10.9	24.1 ± 12.3	0.001
Urea (mg/dL)	23.6 ± 16.2	23.1 ± 17.5	23.2 ± 17.2	25 ± 13.7	22.8 ± 14.9	0.4
Creatinine (mg/dL)	0.8 ± 0.3	0.75 ± 0.3	0.76 ± 0.2	0.77 ± 0.2	0.76 ± 0.2	0.6
SUA (mg/dL)	4.7 ± 1.3	4.5 ± 1.5	4.7 ± 1.2	4.6 ± 1.4	4.9 ± 1.5	0.04
Lipid profile:						
Cholesterol (mg/dL)	197.1 ± 65.8	192.6 ± 67.9	204.4 ± 61.7	169.2 ± 78.9^a,b^	199.6 ± 54.9^c^	<0.0001
LDL (mg/dL)	102.3 ± 40.7	105.3 ± 44.2	107.1 ± 36.6	90.0 ± 47.7	100.9 ± 33.2	<0.0001
HDL (mg/dL)	56.7 ± 30.9	58.4 ± 33.8	56.6 ± 27.8	53.5 ± 34.2	60.7 ± 32.2	0.1
Triglycerides (mg/dL)	122.6 ± 55.8	117.7 ± 61.1	137.3 ± 52.5^a^	98.5 ± 55.2^a,b^	112.6 ± 43.4^b^	<0.0001

DNRA, Double seronegative RA; DPRA, Double seropositive RA; RF, Rheumatoid factor; Anti-CCP, Anti-cyclic citrullinated peptide; Hb, Hemoglobin; TLC, Total leucocyte count; ESR, Erythrocyte sedimentation rate; CRP, C-reactive protein; ALT, Alanine aminotransferase; AST, Aspartate aminotransferase; SUA, Serum uric acid; LDL, Low-density lipoprotein; HDL, High-density lipoprotein; ANA, Antinuclear. Statistically significant p values are in bold. ^a^Significantly different from DNRA, ^b^Significantly different from DPRA, ^c^Significantly different from RF+/anti-CCP-.

**Table 4:** Characteristics of rheumatoid arthritis patients and comparison of different groups regarding the medications use according to RF and anti-CCP autoantibody status

Parameter Mean ±SD or *n* (%)	Rheumatoid arthritis patients					

			Seropositive rheumatoid arthritis (n=4389)			
	
	All (*n* = 5268)	DNRA (*n* = 879) 0	DPRA (*n* = 2900) 1	RF+/anti-CCP- (*n* = 892) 2	anti-CCP+/RF- (*n* = 597) 3	*P* value
Steroids	3104/4425 (70.2)	440/646 (68.1)	1849/2560 (72.2)	468/722 (64.8)	347/497 (69.8)^b^	0.01
Methotrexate	3383/4525 (74.8)	494/663 (74.5)	1984/2606 (76.1)	510/737 (69.2)^b^	395/519 (76.1)^c^	0.002
Leflunomide	2024/4005 (50.5)	306/543 (56.4)	1160/2376 (48.8)^a^	342/654 (52.3)	216/432 (50)	0.01
Sulfasalazine	156/434 (35.9)	28/47 (59.6)	13/37 (35.1)^a^	23/42 (54.8)^b^	13/37 (35.1)	<0.0001
Cyclophosphamide	43/2014 (2.1)	5/282 (1.8)	27/1219 (2.2)	9/342 (2.6)	2/171 (1.2)	0.7
Azathioprine	48/2237 (2.2)	9/301 (2.9)	30/1372 (2.2)	7/383 (1.8)	2/181 (1.1)	0.5
Mycophenolate mofetil	11/1984 (0.5)	1/276 (0.4)	5/1203 (0.4)	5/338 (1.5)	0/176 (0)	0.08
Cyclosporine A	5/1863 (0.3)	0/241 (0)	4/1185 (0.3)	0/298 (0)	1/139 (0.7)	0.4
Biologics	354/3095 (11.4)	46/401 (11.5)	225/1895 (11.9)	57/477 (11.9)	26/322 (8.1)	0.3
Colchicine	35/1875 (1.9)	8/247 (3.2)	19/1188 (1.6)	4/299 (1.3)	4/141 (2.8)	0.2

DNRA, double seronegative RA; DPRA, double seropositive RA; RF, rheumatoid factor; Anti-CCP, anti-cyclic citrullinated peptide; HCV, hepatitis C virus. Statistically significant *P* values are in bold. ^a^Significantly different from DNRA, ^b^Significantly different from DPRA, ^c^Significantly different from RF+/anti-CCP-.

Table 5 summarizes the multinomial logistic regression. Patients with older age (RRR: 1.03, 95%CI: 1.0, 1.0, *P* = 0.029), greater DAS28 (RRR: 1.51, 95%CI: 1.2, 1.9, *P* < 0.001), higher steroid use (RRR: 2.4, 95%CI: 1.36, 4.25, *P* = 0.002) were at higher odds of DPRA, while, subjects with lower MTX (methotrexate) (RRR: 0.29, 95%CI: 0.13, 0.65, *P* = 0.003) and leflunomide (RRR: 0.56, 95%CI: 0.31, 0.99, *P* = 0.046) use were at lower risk of falling into the DPRA group. Subjects with longer disease duration (RRR: 1.08, 95%CI: 1.01, 1.16, *P* = 0.017), and FMS (fibromyalgia) (RRR: 2.54, 95%CI: 1.10, 5.88, P= 0.028) were associated with higher odds of RF+/ anti-CCP-status.

**Table 5:** Multinomial logistic regression analyses of positive RF and/or anti-CCP as a risk factor among patients with RA in ECR-RA

Variables	Double positive		RF+/anti-CCP-		RF-/anti-CCP+	
		
	RRR (95%CI)	*P* value	RRR (95%CI)	*P* value	RRR (95%CI)	*P* value
Double negative	Ref		Ref		Ref	
Age	1.03 (1.00, 1.05)	0.029	1.00 (0.98, 1.03)	0.677	1.01 (0.98, 1.04)	0.615
Sex (Male)	1.59 (0.64, 3.97)	0.311	0.87 (0.29, 2.63)	0.806	1.48 (0.51, 4.27)	0.465
Disease duration	1.06 (0.99, 1.12)	0.077	1.08 (1.01, 1.16)	0.017	0.98 (0.91, 1.07)	0.680
Smoking	1.62 (0.29, 9.18)	0.584	3.91 (0.59, 26.10)	0.159	1.76 (0.25, 12.56)	0.573
Arthritis	0.87 (0.38, 2.07)	0.744	0.74 (0.31, 1.75)	0.492	0.63 (0.25, 1.61)	0.338
Ocular	1.57 (0.55, 4.50)	0.399	1.59 (0.51, 4.98)	0.424	1.23 (0.33, 4.5)	0.757
CVS	1.08 (0.37, 3.17)	0.891	0.42 (0.10, 1.74)	0.232	1.44 (0.39, 5.29)	0.581
FMS	1.88 (0.84, 4.20)	0.122	2.54 (1.10, 5.88)	0.028	0.92 (0.32, 2.63)	0.882
MetS	6.02 (0.74, 48.67)	0.092	4.22 (0.46, 38.78)	0.204	1.05 (0.06, 18.03)	0.975
DAS28	1.51 (1.20, 1.90)	<0.001	1.16 (0.90, 1.49)	0.240	1.10 (0.84, 1.45)	0.491
Steroid use	2.41 (1.36, 4.25)	0.002	0.93 (0.48, 1.78)	0.831	0.99 (0.49, 2.02)	0.990
MTX use	0.29 (0.13, 0.65)	0.003	0.49 (0.20, 1.20)	0.119	0.50 (0.19, 1.33)	0.166
LFN use	0.56 (0.31, 0.99)	0.046	1.16 (0.59, 2.26)	0.661	0.93 (0.45, 1.90)	0.842
Anemia	1.54 (0.86, 2.74)	0.143	1.30 (0.68, 2.46)	0.420	0.99 (0.49, 2.01)	0.998
PLT	1.00 (0.98, 1.00)	0.703	0.99 (0.99, 1.00)	0.476	1.00 (0.99, 1.00)	0.233

RA, rheumatoid arthritis; RF, rheumatoid factor; Anti-CCP, anti-cyclic citrullinated peptide; RRR, relative risk ratio; 95%CI, 95% confidence interval; CVS, cardiovascular; FMS, fibromyalgia; MetS, metabolic syndrome; DAS28, disease activity score 28; MTX, methotrexate; LFN, leflunomide; PLT, platelets. Statistically significant *P* values are in bold.

## Discussion

The field of autoantibodies has brought major insights in rheumatology. It resulted in better understanding of the pathophysiology in RA and the development of the new 2010 ACR/ EULAR classification for RA. Although autoantibodies, RF and anti-CCP, have prognostic significance in RA;^[4]^ their relation to other disease characteristics remains a debate. The disease phenotype in seronegative and seropositive RA has been previously presented; though comparing groups in view of single-positive serology (RF or anti-CCP), double-negative and double-positive serology has not been thoroughly examined. This work is a large multicentre observational study including 5268 adult RA patients who had results of RF and anti-CCP available, recruited from the ECR-RA study database. A key objective was to compare demographic, clinical, laboratory features, disease activity and severity of Egyptian RA patients across RA groups with different autoantibodies status (DPRA, DNRA, single positive RF and anti-CCP).

In the current study, patients with DPRA clearly showed higher disease activity and severity in regards to those with dual negative antibodies status. Additionally, markers of systemic inflammation including ESR, CRP, platelet count were significantly higher in patients with DPRA versus others, denoting more aggressive disease. In line, high disease activity and impaired physical function were reported in RF+/anti-CCP+ versus seronegative RA patients.^[9]^ In consistency, DAS-28, ESR and CRP were significantly higher in patients with DPRA versus DNRA, single positive RF and anti-CCP.^[16]^ Anti-CCP is associated with greater disease activity and poorer remission rates.^[17]^ The DAS-28 was comparable among seronegative (double negative for RF and anti-CCP) and seropositive patients; however, more joint damage was reported with seropositive RA.^[18]^ Conversely, significantly higher DAS-28, HAQ score, more radiographic progression and slower response to treatment were reported in negative RF/anti-CCP versus positive RF/anti-CCP.^[19]^ Conflicting results are reported regarding the impact of RF versus anti-CCP positivity on disease activity; anti-CCP positive patients had lowest disease activity and disability regardless RF status; and achieved faster remission.^[20]^

The presence of autoantibodies is a distinctive feature of RA. Several studies have indicated the clinical relevance of RF and anti-CCP antibodies in RA. High titers of RF have been associated with more aggressive articular disease, and higher prevalence of extra-articular manifestations, especially when in combination with ACPA.^[16,21,22]^ In the present study, ocular manifestations were found to be higher in patients with DNRA (22.3%). In previous studies, there was no relationship between RF and ocular surface involvements.^[23,24]^ However, there was a significant association between the presence of anti-CCP and ocular manifestations related to RA.^[25]^ Patients with either positive anti-CCP or RF presented with higher fibromyalgia syndrome, which is also indicative of clinical active disease. Positivity of RF and anti-CCP was evaluated between patients with or without FMS in RA patients. Rheumatoid factor was positive in 67.9% and 75.5% and anti-CCP was positive in 71.7% and 76.5% of the patients with or without FMS, respectively (*P* > 0.05).^[26]^ The current study indicates that there is no significant association between neurological manifestations in patients with RA and autoantibodies positivity. In some cases, but not all, the presence of ACPA and RF autoantibodies can be detected in the pathologic analysis of neurologic lesions in patients with RA.^[27]^ In the current study, presence of mucous ulcer was significantly higher in patients with anti-CCP antibodies. Recent data are suggestive of a role for mucosal surfaces in the development of auto-immune responses (autoantibodies production) associated with the development of RA.^[28]^ Indeed differences in RA-patients with and without RF) and anti-CCP have been observed such as differences in clinical presentations.

In the current work, patients with single positive anti-CCP were significantly younger and had an earlier age of disease onset compared to other groups. Significant association between gender and seropositivity was found with female pre-dominance across all groups. On regression, older age was significantly associated with DPRA. It is widely accepted that RA encompasses great heterogeneity. Both antibodies may represent different RA diseases with specific courses and drug responsiveness. Therefore, RF and anti-CCP cannot be used synonymously or interchangeably as they represent different disease entities.

In the current work, MetS was the most prevalent comorbidity in DPRA (19.3%). On comparing seronegative and sero-positive RA patients, hypertension, type II diabetes and dyslipidemia were the most frequent comorbidities and similar. ^[29]^ Currently, significant association was found between the type of RA and comorbidities in which MetS was significantly more frequent in DPRA. In addition, the extra-articular manifestations that often determine the severity and comorbidity of RA are also closely associated with anti-CCP positivity.^[30]^ Both anti-CCP and RF have been found to associate with cardiovascular disease and mortality in RA patients.^[31]^ In contrast to the current work, others showed akin results on comparing seronegative and seropositive RA regarding co-morbidities.^[19]^ The traditional cardiovascular disease (CVD) risk factors were significantly associated with single positive RA, in which BMI, LDL (low-density lipoprotein), cholesterol and triglycerides were significantly higher in DPRA patients.

However, surprisingly CVD occurred in 14.5% of double negative RA which was significantly higher compared to other groups. In contrast, no significant difference was found on comparing seropositive and seronegative RA regarding the 10-year CVD risk.^[18]^ It has been proposed that the autoantibodies might contribute to CVD burden; in which anti-CCP antibodies have been associated with long-term mortality and worse CVD outcomes in non-RA patients.^[32]^ However, the heightened inflammatory state could accelerate the process of atherosclerosis and subsequent CVDs.^[33]^ The traditional CVD risk factors account only for 50% of total CVD risk in RA patients;^[34]^ implying the strong interplay between inflammation, atherogenesis and CVDs.

As already stated before, RFs are detectable in non-rheumatic conditions as well. The frequency of RF positive individuals in infectious diseases depend on whether it is a primary or secondary infection. In the current work, HCV infection was significantly more frequent in single positive RF patients. In line, 50%–70% of patients with HCV infection have positive RF antibodies, while anti-CCP was not observed; limiting the diagnostic utility of RF in HCV-related arthropathy.^[35]^ The estimated prevalence for HCV globally is 3%;^[36]^ however, exceptionally in Egypt, it is estimated to be 10%–15%.^[37]^ Lymphotropism which is a prominent feature of HCV-infection is characterized by stimulation of the immune system resulting in autoantibodies production as RF and cryoglobulins.^[38]^

The significance of the current work is boosted by the fact that it is among few studies investigating the effect of concordant presence of both RF anti-CCP and its impact on disease phenotype. The autoantibodies have several pathogenic potentials through immune complex formation and their binding to Fc receptor on the macrophages which promotes subsequent tumor necrosis factor (TNF) production;^[39]^ and through complement activation.^[40]^ Additionally, anti-CCP antibodies stimulate production of neutrophil extracellular traps (NETs) which trigger a proinflammatory state through enhancing production of cytokines and adhesion molecules in RA.^[41]^ Interestingly, immune-complex assays showed that adding RF IgM boosted the capacity of the anti-CCP to stimulate the macrophages resulting in increased cytokines production,^[16]^ highlighting the integration of both autoantibodies in RA inflammatory status. In line, the presence of several autoantibodies has been associated with an augmented inflammatory response in RA.^[42]^ It has been proposed that seronegative RA might require less aggressive treatment ^[43]^ and is not a mild disease phenotype.^[10,19]^

The cross-sectional study design is a limitation to find out a casual-relationship between the autoantibody status, disease characteristics and response to treatment. Liver function tests were significantly higher in patients with RF+/anti-CCP+ autoantibody status, which could be attributed to the greater use of methotrexate and leflunomide in DPRA. However, this is a leading large multicentre real-world study of Egyptian RA patients capable to detect small differences among different groups. Results need to be validated through larger scale longitudinal studies to prove causality. Additionally, different measures of disease activity as the clinical disease activity score were not explored; and no data have been collected regarding structural joint damage.

In conclusion, all the data together brought light on comparing different groups of RA patients based upon RF and anti-CCP autoantibody status. Patients with double seropositivity (RF+/ anti-CCP+) had more severe disease phenotype evidenced by the significantly increased markers of inflammation, increased frequency of arthritis, greater use of steroids and the more impairment in physical function. Current guidelines for initial therapy still do not promote to treat seropositive and seronegative RA differently. Considering tailored therapy for patients with both RF and anti-CCP may allow for better outcomes. However, in the current study CVD was significantly more frequent in DNRA in spite of the association of several traditional CVD risk factors with RF+/anti-CCP+, highlighting the considerable inflammatory and clinical burden of RF-/ anti-CCP-which might provide a better definition for this disease phenotype. This leaves an interesting area for further research, aiming to gain more knowledge.
